# Ecological associations between ambient air pollution, gut microbiome composition, and metabolic markers in urban populations of central India

**DOI:** 10.3389/fpubh.2026.1834285

**Published:** 2026-09-14

**Authors:** Mona Kriti, Raj Ojha, Samradhi Singh, Devojit Kumar Sarma, Vinod Verma, Manoj Kumawat, Anita Tilwari, Rajnarayan Tiwari, Manoj Kumar

**Affiliations:** 1 ICMR–National Institute for Research in Environmental Health, Bhopal, Madhya Pradesh, India; 2 Department of Microbiology, Barkatullah Vishwavidyalaya, Bhopal, Madhya Pradesh, India; 3 Stem Cell Research Centre, Department of Hematology, Sanjay Gandhi Post-Graduate Institute of Medical Sciences, Lucknow, Uttar Pradesh, India; 4 Department of Biological Sciences, Indian Institute of Science Education and Research Bhopal (IISERB), Bhopal, Madhya Pradesh, India

**Keywords:** air pollution, gut microbiome, NO_x_, PM_10_, PM_2.5_, Type 2 diabetes (T2D)

## Abstract

**Objective:**

To investigate the ecological level associations and spatial co-variation among ambient air pollutants, Type 2 diabetes (T2D) related clinical markers, and gut microbiome composition in urban populations of Bhopal, India.

**Methods:**

A cross-sectional ecological study was conducted involving 95 participants from three urban locations in Bhopal. Ambient air quality data, including PM_2.5_, PM_10_, NO_x_, and SO_2_, were obtained from local monitoring stations. Exposure assessment was based on location level ambient monitoring data corresponding to participants’ residential localities rather than individual level personal exposure measurements. Clinical assessments were performed to measure T2D related metabolic parameters, and fecal samples were analyzed using full-length 16S rRNA sequencing to profile gut microbiome composition. Associations between pollutant levels, metabolic markers, and microbial taxa were evaluated using exploratory correlation and compositional analyses.

**Results:**

Variability was observed in both air pollutant concentrations and metabolic parameters. NO_x_ levels were positively correlated with glycemic indices, while particulate pollutants (PM_2.5_ and PM_10_) showed inconsistent and non-directional associations with metabolic markers. Gut microbiome analyses revealed broadly similar microbial community structures with subtle differences in diversity and composition across locations. Although variations were observed in selected microbial taxa, most genus-level differences did not remain statistically significant following multiple testing correction.

**Conclusion:**

Air pollution and gut microbiome composition appear to have a descriptive role in metabolic health. The findings describe ecological level co-variation between ambient air pollution indicators, metabolic parameters, and gut microbiome composition across the study populations. Because the study design was cross-sectional and exposure assessment relied on location level monitoring data, the results should be interpreted as descriptive and hypothesis-generating rather than causal. Further longitudinal studies incorporating individual level exposure assessment and functional microbiome analyses are required to better evaluate these relationships.

## 1 Introduction

The global epidemiological landscape is undergoing a significant transition due to socioeconomic development, urbanization, and demographic shifts, with air pollution emerging as a leading environmental health risk worldwide. Exposure to ambient pollutants, particularly fine and ultrafine particulate matter, has been linked to elevated rates of cardiovascular, respiratory, and metabolic diseases, including Type 2 diabetes mellitus (T2D), contributing substantially to morbidity and mortality (1–3). India, with both high ambient pollution levels and rapidly rising diabetes prevalence, represents a particularly vulnerable setting, where surveys indicate a T2D prevalence of 11.4% and prediabetes affecting 15.3% of adults (4). Rapid urbanization in India has intensified industrial activities, vehicular traffic, and energy consumption, all contributing to deteriorating air quality, and air pollution was estimated to account for 1.67 million deaths in India in 2019 (5–7). Studies reported an increase in India’s average annual PM_2.5_ levels of 27 μg/m^3^ in 1998 to 44 μg/m^3^ in 2022 in major cities like Delhi and Mumbai with Chennai experiencing the highest concentrations (8). Epidemiological evidence further links PM_2.5_ exposure to elevated fasting glucose, HbA1c, and T2D incidence, while PM_10_ has been associated with higher HbA1c, insulin resistance, and postprandial glucose levels, particularly among young-onset diabetes patients (9, 10). The International Diabetes Federation estimated that around 451 million individuals got diabetes in 2017, a figure expected to climb to 693 million by 2045, with the most rapid growth anticipated in regions of Africa, the Pacific, and Asia (3, 11, 12).

Biological mechanisms connecting air pollution and T2D include systemic inflammation, oxidative stress, endothelial dysfunction, epigenetic modifications and disturbances in glucose metabolism (13–15). Emerging evidence also implicates the gut microbiome as a potential mediator, given its central role in host metabolism, immune regulation, and short-chain fatty acid (SCFA) production (16). Experimental studies also support the role of the gut microbiome as an associated factor linking air pollution to metabolic disease. For instance, animal studies have demonstrated that ingestion or inhalation of particulate pollutants alters gut microbial composition, including shifts in *Bacteroidetes*, *Firmicutes*, and *Verrucomicrobia*, accompanied by changes in SCFA production and impaired glucose tolerance (17, 18). Additionally, human studies have reported reduced abundance of butyrate-producing bacteria such as *Faecalibacterium prausnitzii* and *Roseburia* spp. in individuals with T2D (19). Similarly, a pilot study among obese adolescents demonstrated that traffic-related air pollution exposure was associated with gut microbial alterations and fasting glucose levels, with microbial functional changes explaining over 20% of the association (20). Study from China using the Guangdong Gut Microbiome Project dataset have further demonstrated significant associations between gut microbiome diversity and T2D, alongside marked heterogeneity in PM exposure within the region. Notably, recent investigations suggest that gut microbial dysbiosis may mediate associations between PM exposure especially PM_2.5_ and PM_1_ with impaired fasting glucose and T2D, supporting the possibility of microbiome-related associations (21, 22). Nevertheless, most studies have overlooked the prediabetes stage and have relied on self-reported data, particularly in low-resource urban Indian populations (23–25). Given the gap, along with rapidly increasing prevalence of diabetes and the unique environmental and dietary contexts in India, the present study aims to investigate the association between chronic ambient air pollution exposure and T2D prevalence in an urban population of central India. Specifically, we investigate whether gut microbiome composition and diversity co-vary with ambient pollution indicators and metabolic profiles.

## 2 Materials and methods

### 2.1 Ethics

All study procedures were conducted in full compliance with the ethical guidelines approved by the Institutional Ethics Committee (IEC) of ICMR-NIREH under approval number NIREH/BPL/IEC/2024–25. Additionally, a written informed consent was obtained from each subject before enrollment.

### 2.2 Subject enrolment and categorization

Sample collection was carried out in Bhopal, Madhya Pradesh, India, through a survey of over 300 households. Adults aged 18–65 without major health issues were eligible, and 95 participants voluntarily provided blood and fecal samples after giving informed consent. Participants were classified in diabetic, prediabetic and healthy controls according to their HbA1c levels based on the American Diabetes Association (ADA) criteria. Participants with HbA1c ≥ 6.5% were classified as diabetic (T2D), individuals with HbA1c between 5.7 and 6.4% were categorized as prediabetic (PD), and participants with HbA1c < 5.7% were categorized as healthy controls (Ctrl). Information regarding dietary preferences, smoking status, alcohol consumption, socioeconomic status, and physical activity was collected through participant questionnaires where available.

Participant categorization and pollution exposure assignment were based on residential locality at the time of recruitment. Ambient air pollution exposure was estimated using the nearest CPCB monitoring station corresponding to each participant’s residential area rather than through direct personal exposure monitoring. Therefore, the exposure estimates represent location level ambient exposure proxies and do not account for individual variability in commuting behavior, occupational exposure, residential history, indoor air pollution, or daily mobility patterns.

### 2.3 Clinical parameters

Serum samples were prepared by allowing blood collected in plain glass vials to clot, followed by centrifugation. Key clinical parameters including serum creatinine, triglycerides, total cholesterol, and fasting insulin were subsequently analyzed. Glycated A1c (HbA1c) and fasting blood glucose (FBS) levels were assessed on-site using finger-prick method.

Participants were instructed to maintain overnight fasting for at least 8–10 h prior to blood glucose assessment. Internal quality control procedures were conducted according to manufacturer guidelines before sample analysis, and all measurements were obtained using calibrated instruments operated by trained personnel under standardized field conditions.

Anthropometric data, including height, weight, waist circumference, and hip circumference, were recorded to calculate indicators such as Body Mass Index (BMI), waist-to-hip ratio (WHR), and waist-to-height ratio (WHtR). Furthermore, insulin function and sensitivity were evaluated using indices like the Homeostatic Model Assessment for *β*-cell function and Insulin Resistance (HOMA-B and HOMA-IR), along with the Quantitative Insulin Sensitivity Check Index (QUICKI).

### 2.4 Sample preparation and sequencing

Freshly collected fecal specimens were immediately preserved at −80 °C in the institutional biorepository and processed for metagenomic DNA extraction within a median interval of three days. High-quality microbial DNA was isolated using the QIAGEN DNeasy PowerSoil Pro Kit, strictly following the manufacturer’s protocol. DNA yield and purity were assessed prior to downstream applications. Full-length 16S rRNA gene amplification (V1–V9) was carried out using PacBio-compatible universal primers. Library construction was performed using the SMRTbell Express Template Preparation Kit 2.0, with 1.7 μg of purified PCR amplicons as input material. The resulting SMRTbell libraries had an average insert size of 1,903 bp. Sequencing was conducted on the PacBio Sequel II platform using the Sequel II Binding Kit 2.1, with 90 pM of the prepared library loaded onto an 8 M SMRT Cell. This sequencing run generated a mean of 872,446 subreads, with an average read length of 1,523.78 bp across samples.

### 2.5 Data collection, processing, and statistical analysis

Data on air pollution were obtained from certified CPCB (Central Pollution Control Board) environmental monitoring stations in all the three locations, namely Bairagarh (Bai) (LAT: 23.2719993146122°N, LON: 77.3404630659457°E), Hoshangabad Road (Hos) (LAT: 23.2031026231466°N, LON: 77.4459231947791°E), and Mrignayani (Mri) (LAT: 23.2632988847620°N, LON: 77.4109275056545°E) from Bhopal, Madhya Pradesh, India (Figures 1A–E). The pollutants analyzed include PM_10_, PM_2.5_, NO_x_, and SO_2_.

**Figure 1:**
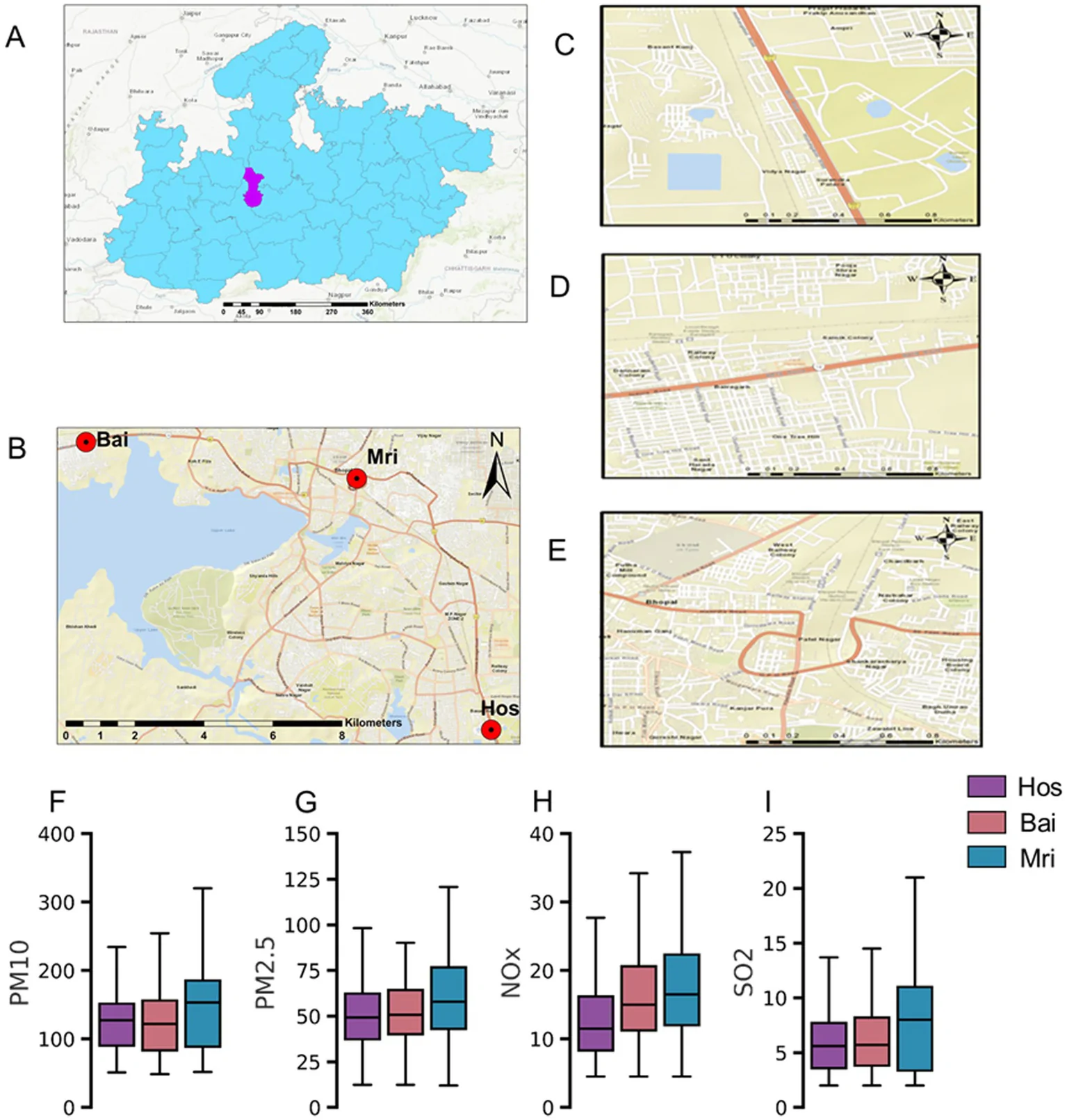
Assessment of air pollution indicates significant differences between the daily pollutant concentrations of the locations **(A)** Location of Bhopal in Madhya Pradesh, India. **(B)** Location of the three study localities in Bhopal: Hos, Bai, and Mri. **(C–E)** Maps showing the individual study localities: **(C)** Hos, **(D)** Bai, and **(E)** Mri. Maps were generated using ArcGIS Desktop 10.8.1. **(F–I)** Distribution of ambient air pollution concentrations across the three study localities: **(F)** PM_10_, **(G)** PM_2.5_, **(H)** NO_x_, and **(I)** SO_2_.

Exposure estimates were derived from location-level ambient monitoring station data corresponding to participants’ residential localities. Five-year averaged pollutant concentrations were used to characterize spatial variation between locations. Therefore, the exposure window reflects long-term ecological averages rather than participant-specific personal exposure histories. Because individual residential history, commuting behavior, occupational exposure, indoor exposures, and daily mobility patterns were not available, exposure misclassification cannot be excluded and have been considered while interpreting the findings.

Descriptive statistical analyses were conducted, including calculation of mean, median, standard deviation, skewness, kurtosis, and coefficient of variation (CV). Additionally, Kruskal-Wallis test was performed to compare the differences between the locations and missing data was handled by skipping them from the analysis. Spearman’s correlation was used for clinical association analysis, significance was set at *p* < 0.05 and coefficients were calculated to explore the strength and direction of relationships between pollutant concentrations and clinical parameters.

Microbiome sequencing reads were analyzed using the QIIME2 platform (version 2023.5). Initial processing included demultiplexing and quality control through the q2-demux plugin. Sequence dereplication, error correction, and chimera filtering were carried out using DADA2-CCS, specifically adapted for single-end PacBio circular consensus sequences. Analyses were performed using default settings: sequences were required to include the forward primer (permitting up to two mismatches), primers were trimmed automatically, no fixed-length truncation was imposed, quality filtering was based on a maximum expected error threshold of 2.0, denoising was executed separately for each sample, and chimeras were removed using a consensus-based approach according to standard QIIME2 guidelines. Resulting amplicon sequence variants (ASVs) were aligned using MAFFT. Taxonomic classification was assigned with a scikit-learn–based naïve Bayes classifier trained against the SILVA 138 reference database at 99% sequence similarity.

Following taxonomic classification, genus-level feature tables were generated for downstream analyses. Alpha diversity indices, including Observed richness, Chao1, Shannon index, Simpson index, Rényi diversity, and Heip evenness, were calculated at the genus level and compared across groups using the Kruskal–Wallis test. Beta diversity was evaluated using Bray-Curtis and Jaccard distance matrices computed at the genus level. PCoA was performed to visualize community dissimilarities, and the proportion of variance explained by the first two components was reported.

For genus-level comparisons, taxa detected in more than 50% of samples were retained for statistical testing. Group-wise differences were assessed using the Kruskal-Wallis test, and *p*-values were adjusted using False Discovery Rate (FDR) correction to account for multiple comparisons. Because most genus-level differences did not remain statistically significant following FDR correction, these findings were interpreted cautiously as exploratory compositional trends rather than definitive microbial signatures. Shared and unique genera count across the three locations were identified using genus level dataset, and Venn diagrams were constructed using InteractiVenn to visualize overlapping and location specific taxa (26).

Additionally, principal component analysis (PCA) was conducted using city-level averages of microbial genera, metabolic parameters, and air pollution variables to explore integrated spatial patterns and hence the analysis should be interpreted as exploratory and descriptive rather than inferential. All statistical analyses were performed using Python (version: 3.11).

## 3 Results

### 3.1 Assessment of air pollution indicates significant differences between the daily pollutant concentrations of the locations

Since late 2017, PM_10_ has consistently recorded the highest concentrations among pollutants, with PM_2.5_ ranking second. However, NO_x_ and SO_2_ were static and less in concentrations. This implies to PM_10_ being a major pollutant in terms of concentrations polluting the air composition of the studied locations. The drops in the concentrations of the pollutants in between the year can be accounted as the effect of rainfalls in monsoon season and the locations were consistent with the aforementioned trends (Supplementary Figure S1). However, PM_10_ concentrations were significantly higher at the Mri location compared to the others, as was the case for PM_2.5_ and SO_2_. Interestingly, Hos exhibited a distinct profile for NO_x_, yet Mri remained the most polluted site overall (Figures 1F–I). These patterns highlight the spatial and temporal variation in location-level ambient pollutant concentrations; however, they should not be interpreted as direct measures of individual participant exposure because personal exposure variability was not assessed.

### 3.2 Clinical comparisons and their associations with pollutants and disease prevalence

Using the Kruskal–Wallis test, significant differences were observed in the TyG index, cholesterol, pulse pressure (PP), systolic blood pressure (SBP), triglycerides (Tg), serum creatinine, mean arterial pressure (MAP), and central systolic blood pressure (cSBP) across the three populations. The TyG index was slightly higher in the Hos population, whereas cholesterol levels were highest in Bai and lowest in Mri. PP was comparable (~50 mmHg) in Hos and Bai but lower in Mri. SBP and MAP were highest in Bai, followed by Hos and Mri. Triglyceride levels were lowest in Bai, while cSBP was highest in Hos (Figures 2A–I). Collectively, these findings indicate distinct cardiometabolic profiles among the three populations.

**Figure 2:**
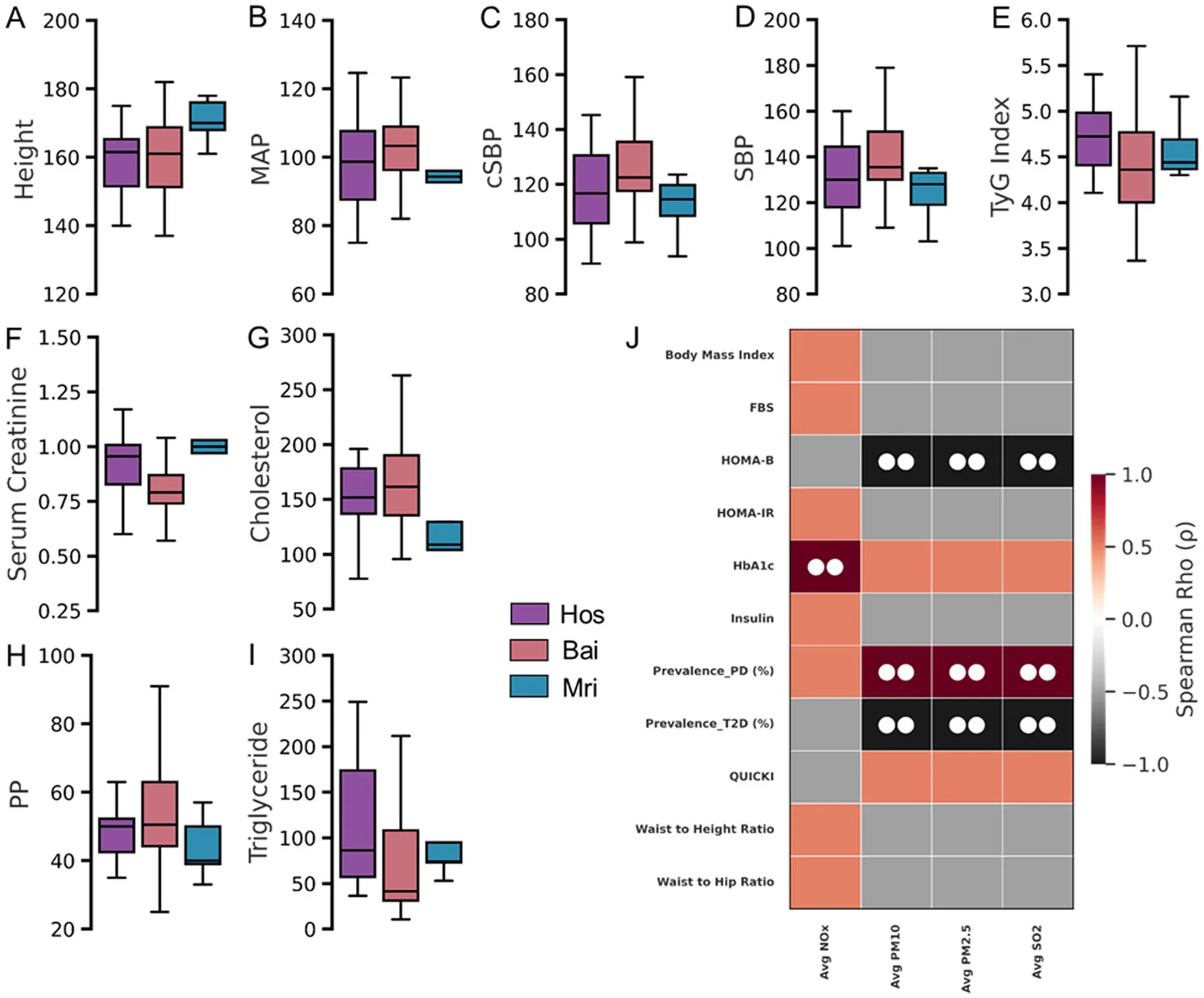
Clinical comparisons and their associations with pollutants and disease prevalence. **(A–I)** Significant comparisons at 90 and 95% CI of the clinical parameters: **(A)** Height, **(B)** MAP, **(C)** cSBP, **(D)** SBP, **(E)** TyG Index, **(F)** Serum Creatinine, **(G)** Cholesterol, **(H)** PP, **(I)** Triglyceride. **(J)** Correlation analyses with *p* < 0.01 relations marked as “●●”.

Spearman correlation analyses performed on location level averaged data demonstrated that the prevalence of PD and HOMA-B were positively correlated with average PM_10_, PM_2.5_, and SO_2_ concentrations, whereas the prevalence of T2D was negatively correlated with these pollutants. Additionally, HbA1c co-varied positively with average NO_x_ concentrations (Figure 2J). Lifestyle and socioeconomic variables including diet, smoking, alcohol use, and physical activity were collected descriptively; however, incomplete availability across participants and the ecological nature of exposure assignment limited formal covariate-adjusted modeling. These analyses represent ecological-level co-variation patterns and should not be interpreted as evidence of direct causal relationships between pollutant exposure and metabolic outcomes.

### 3.3 Compositional subtleties of the gut microbiome exist among locations

As per the classified groups based on localities, the gut microbiota composition was broadly similar among all the groups, with differences observed mainly in the relative proportions of key taxa. Across all groups, the community composition was dominated by *Firmicutes*, which accounted for approximately half of the total relative abundance in each group, with a modest increase from Hos to Bai and Mri. *Actinobacteria* and *Bacteroidetes* were the next most abundant phyla, together comprising a substantial proportion of the remaining community. Smaller contributions were observed from several additional phyla, including *Proteobacteria, Verrucomicrobia, Tenericutes*, and *Cyanobacteria* each representing a relatively minor fraction of the total abundance (Figure 3A). At genus level, *Bifidobacterium*, *Lactobacillus*, and *Prevotella_9* constituted a substantial proportion of the microbial community, indicating their dominance in the samples. More specifically, the Mri group exhibited a higher relative abundance of *Bifidobacterium*, *Faecalibacterium* and *Agathobacter* compared with Hos and Bai, while *Lactobacillus* showed minimal variation across groups. In contrast, Hos and Bai samples demonstrated comparatively higher proportions of *Prevotella_9* and *Blautia* (Figure 3B). These compositional differences were generally modest and should be interpreted cautiously because most genus-level comparisons did not remain statistically significant following FDR correction.

**Figure 3:**
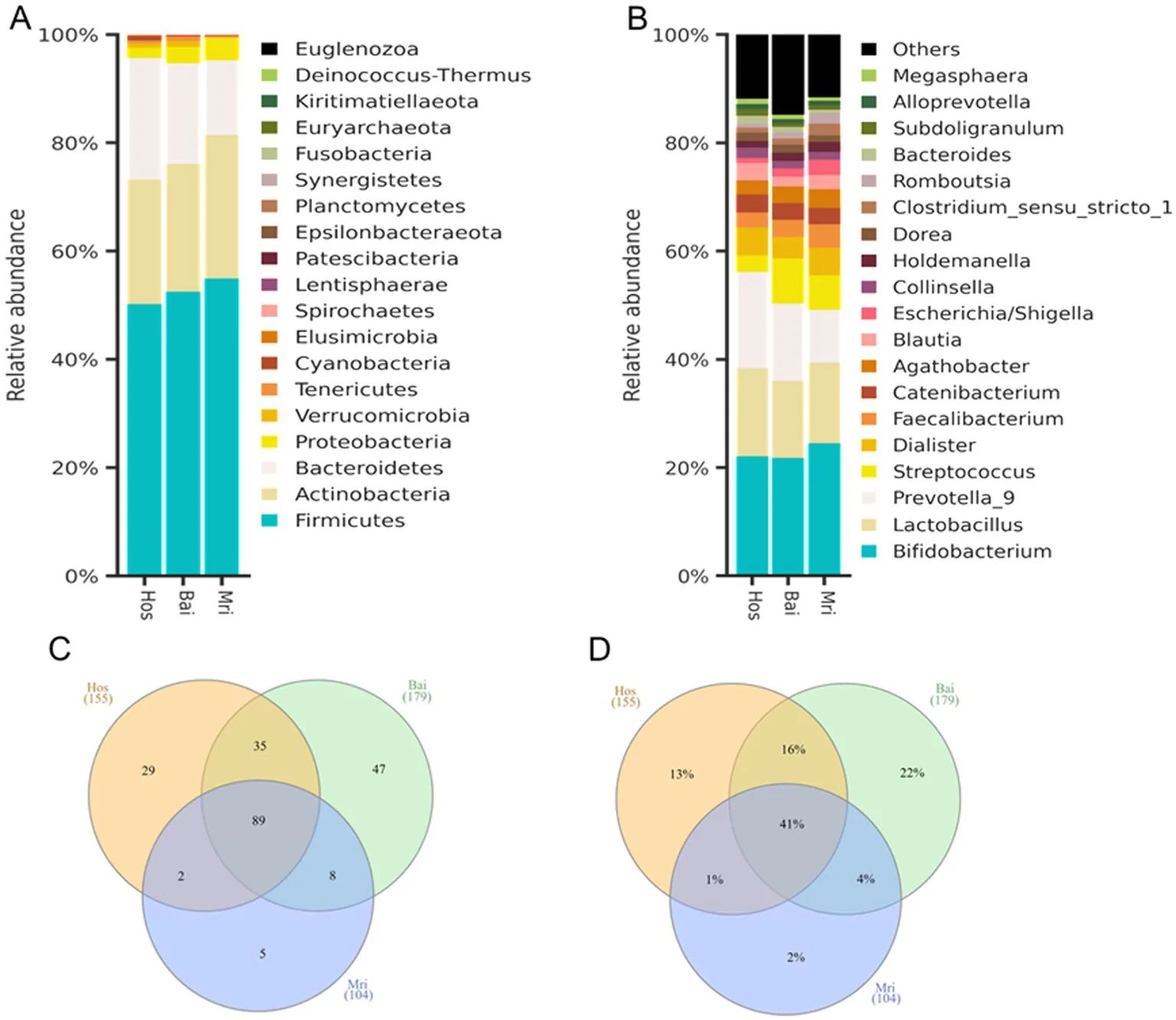
Compositional differences in the gut microbiome. **(A,B)** Relative abundances of top 20 taxa sorted based on abundance sum: **(A)** phylum level, **(B)** genus level. **(C,D)** Venn diagram generated on genus rank: **(C)** counts, **(D)** percentages of composition.

On sample level granulated data, the microbial communities were dominated by a limited number of bacterial phyla, with notable inter-individual variability in their relative proportions. Across all samples, *Firmicutes* and *Bacteroidetes* constituted the majority of the microbial community. *Firmicutes* was the most abundant phylum in most individuals, followed by *Bacteroidetes*, although their relative contributions varied substantially across samples. *Proteobacteria* and *Actinobacteria* were consistently present at lower relative abundances, while phyla such as *Verrucomicrobia, Tenericutes,* and *Cyanobacteria* were detected sporadically. Minor phyla, including *Fusobacteria, Spirochaetes, Synergistetes, Planctomycetes*, *Euryarchaeota*, and *Deinococcus–Thermus,* collectively accounted for a small proportion of the total community. The distribution of these low-abundance phyla differed among individuals but did not dominate any single sample (Supplementary Figure S2A). Across samples at genus level, *Bifidobacterium, Prevotella_9* and *Lactobacillus* were consistently detected at high relative abundances, although their proportional contributions varied markedly among individuals. Additional genera, including *Streptococcus*, *Dialister*, *Faecalibacterium, Agathobacter, Blautia*, and *Escherichia/Shigella*, were present across many samples but generally at lower and more variable relative abundances. Members of the *Ruminococcus, Rikenellaceae RC9 gut group*, and *Megamonas* were detected intermittently, contributing to compositional differences between individuals (Supplementary Figure S2B).

Across all samples, 215 genera were detected, of which 89 genera (41%) were shared among all three localities. The Bai group exhibited the highest number of unique genera (47 genera; 22%), followed by Hos (29 genera; 13%), whereas Mri displayed only 5 unique genera (2%) (Figures 3C,D). The higher number of unique genera observed in the Bai group further supports its greater compositional heterogeneity, consistent with the dispersion patterns observed in beta diversity analyses. In contrast, the limited number of unique genera in Mri aligns with its comparatively tighter clustering pattern in ordination space.

### 3.4 Alpha and beta diversity analyses

Alpha diversity was calculated as well on genus rank and across richness-based metrics, the Mri group exhibited the highest median values for both Observed species and Chao1 indices, indicating greater microbial richness, while the Hos and Bai groups showed lower richness with overlapping interquartile ranges. Diversity indices integrating richness and evenness (Shannon, Rényi, and Simpson) followed a consistent pattern: Mri displayed the highest diversity, Bai was intermediate, and Hos the lowest, with broader variability observed in the Bai and Mri groups. Evenness assessed using the Heip index was comparable between Hos and Bai, albeit with greater dispersion, whereas the Mri group showed a narrower distribution, suggesting a more uniformly structured microbial community (Figures 4A–F). However, none of these differences were statistically significant (Kruskal–Wallis test; *p* > 0.33 for all indices) and therefore these observations should be interpreted as descriptive trends rather than definitive evidence of location-specific microbial diversity differences.

**Figure 4:**
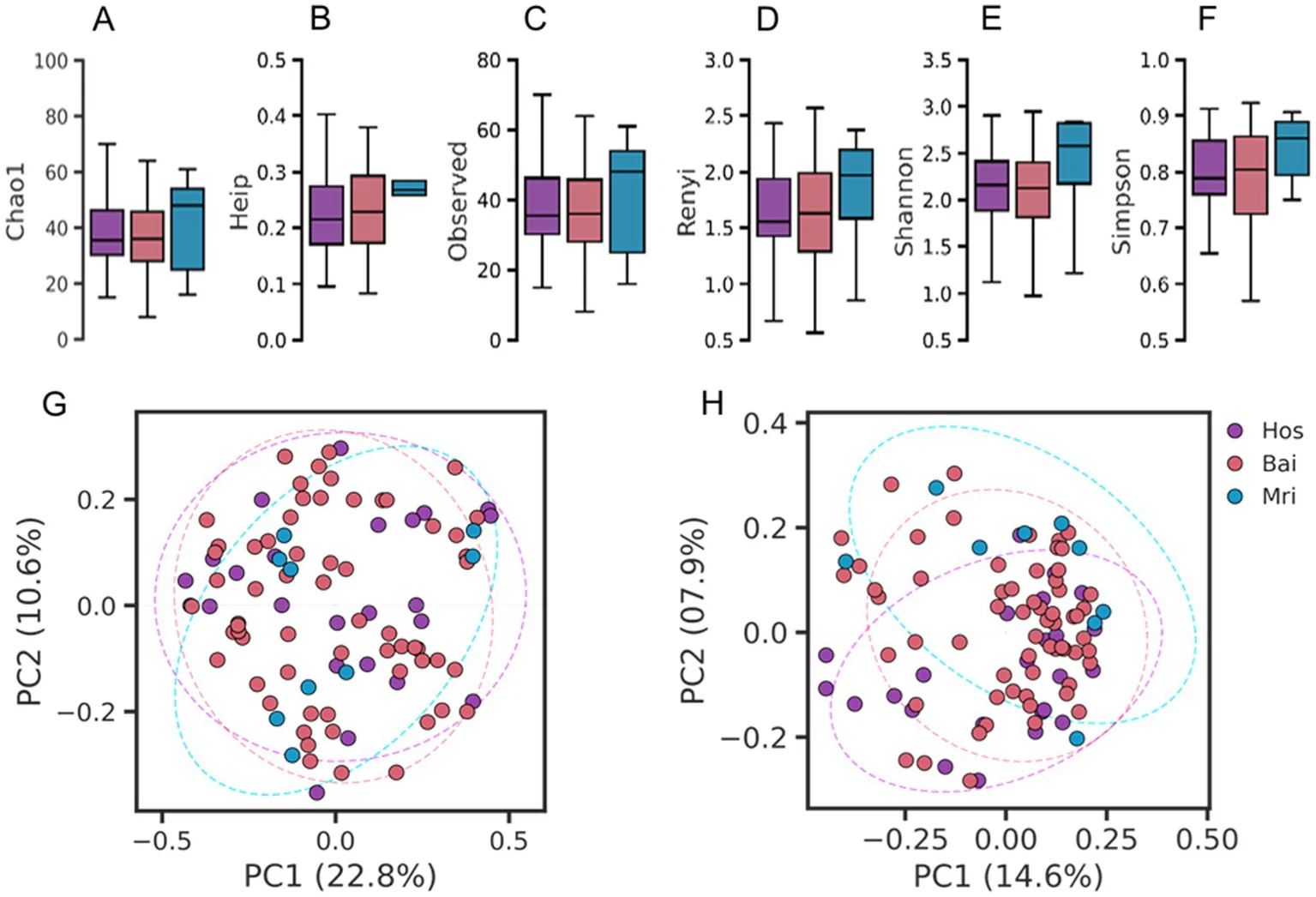
Diversity analyses in the gut microbiome. **(A–F)** Alpha diversity analyses at genus rank with median indicated by a horizontal line. Whiskers extend to the smallest and largest values within 1.5 × IQR from the lower and upper quartiles, respectively **(A)** Chao1, **(B)** Heip, **(C)** Observed, **(D)** Renyi, **(E)** Shannon, **(F)** Simpson. **(G,H)** Beta diversity analyses at the genus level. Dashed ellipses indicate the 95% confidence interval of the ordinated samples **(G)** Bray-Curtis distance matrix, **(H)** Jaccard distance matrix.

Beta diversity was assessed using Bray-Curtis and Jaccard distance matrices calculated at the genus level on raw, non-normalized count data, emphasizing biologically meaningful differences among microbial communities rather than raw sequencing depth.

Bray-Curtis PCoA revealed that the first two components explained 33.4% of the total variance (PC1: 22.8%, PC2: 10.6%). Samples from all three groups (Hos, Bai, Mri) showed substantial overlap, indicating broadly similar global profiles. Nevertheless, group-specific patterns were observed: the Hos group exhibited wider intra-group dispersion, particularly along PC1, suggesting greater heterogeneity; the Bai group clustered moderately near the centroid with a slight shift toward positive PC1; and the Mri group displayed relatively tighter clustering, with samples tending toward negative PC2. 95% confidence ellipses overlapped considerably, but subtle centroid shifts indicate modest group-specific trends (Figure 4G).

PERMANOVA analysis confirmed these observations: there was no significant difference in community composition among groups based on Bray-Curtis distances (pseudo-*F* = 0.669, *p* = 0.846, 999 permutations), whereas Jaccard distances revealed significant differences in community composition between groups (pseudo-*F* = 1.445, *p* = 0.028, 999 permutations). PERMDISP analysis indicated no significant differences in dispersion for either metric (Bray-Curtis: *F* = 0.188, *p* = 0.853; Jaccard: *F* = 0.840, *p* = 0.538), suggesting that the significant differences detected with Jaccard distances reflect true shifts in community composition rather than variability within groups. Jaccard PCoA explained 22.5% of the total variance (PC1 = 14.6%, PC2 = 7.9%). Similar to Bray-Curtis, the three groups partially overlapped, reflecting shared community characteristics. Differences in dispersion and centroid positions were evident: the Bai group showed the largest spread along PC1, indicating higher within-group heterogeneity; the Hos group was concentrated in the lower-left quadrant (negative PC1 and PC2), whereas the Mri group shifted toward the upper-right quadrant (positive PC1 and PC2). 95% confidence ellipses revealed partial separation among groups, with Bai occupying an intermediate position overlapping both Hos and Mri (Figure 4H). Collectively, these analyses indicate that while global gut microbiome profiles are broadly similar across locations, subtle differences in community composition and dispersion patterns are detectable between groups.

## 4 Discussion

The present study reveals spatial differences and ecological-level associations in the distribution of clinical markers related to T2D and their associations with ambient air pollutants across three urban populations. The observed differences in the TyG Index and in cardiometabolic parameters across groups suggest variation in metabolic profiles that co-varied with location-level ambient pollution patterns, particularly air quality indicators. Spearman correlation analyses demonstrated that PD prevalence and HOMA-B were positively associated with PM_10_, PM_2.5_, and SO_2_, whereas T2D prevalence was negatively associated with these pollutants. Additionally, HbA1c showed a positive association with NO_x_ concentrations. Although some of these associations may appear counterintuitive, they should be interpreted with caution. Prior studies have consistently shown that long-term exposure to fine particulate matter has been associated with systemic inflammation, oxidative stress, and impaired glucose metabolism, ultimately increasing T2D risk (26–29). On the other hand, the distinct NO_x_ profile observed in Hos, a location with higher T2D prevalence, may reflect co-occurring spatial trends between traffic-related emissions and metabolic indicators rather than direct causal effects.

However, the majority of pre-existing literature reports positive associations between particulate matter exposure and insulin resistance parameters, with PM generally exerting stronger metabolic effects compared to gaseous pollutants. The inverse association observed between T2D prevalence and particulate pollutants in the present analysis may reflect ecological aggregation, exposure misclassification, or unmeasured confounding factors rather than a protective biological effect (30). Because exposure assessment relied on location-level ambient monitoring data rather than participant-specific personal exposure measurements, the observed associations should be interpreted as descriptive ecological trends only.

Across microbial diversity analyses, only modest differences in gut ecosystem structure emerged among the study groups. The Mri group consistently exhibited higher alpha diversity, characterized by increased richness (Observed and Chao1), greater evenness (Heip), and higher composite diversity indices (Shannon, Simpson, and Rényi). Previous studies have linked some of these genera to SCFA metabolism; however, no functional analyses were performed in the present study within the gut microbiome (31). In contrast, the Hos group showed reduced richness and diversity, along with greater dominance of select taxa, a pattern that may be indicative of altered ecological balance. The Bai group displayed an intermediate and more heterogeneous profile, suggesting transitional microbial states.

At the compositional level, *Bifidobacterium, Lactobacillus, Faecalibacterium*, and *Agathobacter* were observed at relatively higher abundances in the Mri group, whereas *Prevotella_9* and *Blautia* were comparatively more abundant in Hos and Bai groups. Although these genera have previously been discussed in relation to SCFA metabolism and metabolic health in earlier studies, the present study did not directly measure SCFA production, microbial function, inflammatory mediators, or metabolic pathways. Therefore, functional interpretations regarding SCFA production or metabolic effects remain speculative and should not be inferred directly from the current compositional data (31). Conversely, relatively higher abundances of genera such as *Prevotella_9*, *Streptococcus*, and low-level detection of *Escherichia/Shigella* in Hos and Bai groups may reflect ecological shifts influenced by diet, environmental exposures, or host metabolic status, with potential implications for inflammatory tone. Consistent with these observations, previous studies have reported pollution-associated alterations in several of these genera (32–35).

These microbiome patterns gain further relevance when interpreted alongside the observed pollutant–metabolic correlations. Notably, NO_x_, a marker of traffic-related air pollution, showed positive correlations with HbA1c, suggesting a potential link between gaseous pollutants and glycemic regulation. In contrast, particulate pollutants demonstrated more complex associations with *β*-cell function and diabetes prevalence, without consistent directional effects across metabolic parameters. Such patterns may reflect non-linear relationships, or residual confounding, all recognized challenges in air pollution epidemiology. To further explore compositional differences at the genus level, Kruskal-Wallis testing was performed on genera with a detection frequency exceeding 50%. Nominal trends were observed for *Blautia*, *Subdoligranulum*, *Bacteroides*, and *Olsenella*; however, none remained statistically significant following FDR correction. Similarly, Spearman correlation analyses identified positive associations between *Clostridium_sensu_stricto_1* and *Olsenella* with average NO_x_ concentrations. Because these findings did not remain significant after correction for multiple testing, they should be considered exploratory observations rather than robust microbial signatures associated with pollution exposure. Nevertheless, similar pollutant-related associations involving these genera have been reported in previous studies (36–38).

In contrast, particulate pollutants particularly PM_10_, PM_2.5_ and SO_2_ displayed more complex and sometimes counterintuitive relationships with *β*-cell function (HOMA-B), diabetes prevalence, and insulin sensitivity indices. These patterns likely reflect non-linear dose–response relationships, exposure misclassification, population heterogeneity, or compensatory physiological responses, all of which are recognized challenges in air pollution epidemiology. Importantly, these divergent associations underscore that air pollution is not a uniform exposure; rather, individual pollutants exert distinct biological effects through different mechanistic pathways. Accordingly, these findings should not be interpreted as evidence of direct mechanistic effects of specific pollutants on metabolic health outcomes.

When viewed collectively, the data reveal parallel spatial patterns in which air pollution burden, metabolic risk markers, and gut microbial diversity vary across locations. The Hos and Bai groups exhibited lower microbial richness and evenness, suggesting reduced ecological balance, whereas the Mri group showed higher diversity, consistent with a more complex gut ecosystem. However, these observations are associative, and descriptive in nature, and causal relationships cannot be inferred from this cross-sectional ecological-style analysis. Exploratory PCA at the city level broadly reflected these trends, with air pollution indicators co-varying with certain microbial genera and metabolic measures aligning along an orthogonal axis; however, only three city-level observations were included, so these results are descriptive and hypothesis-generating. Overall, the findings suggest possible ecological-level co-variation between environmental exposures, gut microbiota, and metabolic health, providing a foundation for future studies incorporating multi-pollutant models, beta diversity analyses, differential abundance testing, and functional metagenomic inference to investigate mechanistic links.

The inclusion of three distinct, underexplored urban populations allows for a comparative analysis of geographic variability in T2D prevalence and its associated risk factors. By integrating clinical data with ambient air pollution measurements, this study offers a multidimensional perspective on environmental factors associated with metabolic health. However, as a sub-analysis of a larger project, the study is constrained by a moderate sample size, which may limit statistical power to detect associations between ambient air pollution and clinical outcomes such as T2D. The disaggregation of data increased the risk of type II errors, limiting the ability to resolve demographic and environmental diversity within the broader regional population. Additionally, the proximity of the study locations may result in co-localization effects, where participants experience similar environmental exposures, potentially reducing the variability required for robust comparative analyses. Migration and commuting patterns among participants may further influence individual exposure levels to air pollutants, introducing potential misclassification in exposure assessment and affecting the accuracy of observed associations. For comparison, air pollution concentrations and metabolic parameters were averaged over the five-year period for each location, and correlations were conducted using these aggregated data. Because exposure estimates were assigned using location-level ambient monitoring station data rather than direct personal exposure assessment, the analysis is susceptible to ecological fallacy and should not be interpreted as representing individual-level exposure–outcome relationships. Further, adjusted multivariable analyses were not feasible, residual confounding by lifestyle, socioeconomic, and behavioral factors cannot be excluded.

While this ecological-level analysis captures spatial trends, it limits inference about individual-level associations and is susceptible to ecological fallacy. This aggregation may be associated with the unexpected positive correlations and inverse prevalence patterns observed, underscoring the need for caution when interpreting these results and highlighting the importance of future studies employing individual-level exposure and outcome data. Building on the findings of this study, future research should aim to establish causal relationships between air pollution exposure and T2D through longitudinal cohort studies. Incorporating individual-level data such as dietary habits, physical activity, socioeconomic status, medication use, and genetic predispositions would help disentangle the complex interplay between environmental and lifestyle factors in metabolic risk. Expanding the study to include larger and more diverse populations, including geographically more distinct areas with less migration in between them, can enhance the generalizability of results and help identify vulnerable subgroups.

## 5 Conclusion

The study demonstrates differences in air pollution levels, metabolic markers, and gut microbiome composition across three urban populations in Bhopal. Location-level associations and co-variation patterns were observed between specific pollutants and metabolic indices, along with subtle variations in key microbial taxa, across study locations. However, the observed microbiome differences were generally modest, and most genus-level associations did not remain statistically significant after multiple testing correction.

Because the study employed a cross-sectional ecological-style design and relied on location-level ambient monitoring data rather than personal exposure assessment, the findings should be interpreted as descriptive and hypothesis-generating rather than causal. The results highlight potential ecological relationships among air quality indicators, metabolic parameters, and gut microbial composition, while also emphasizing the limitations associated with exposure misclassification and ecological aggregation.

These findings also provide a descriptive foundation for future studies investigating the relationships among air pollution, gut microbiota, and metabolic health. Future investigations incorporating larger cohorts, longitudinal study designs, individual-level exposure assessment, and functional microbiome analyses will be necessary to better evaluate these associations and their potential biological relevance.

## Data Availability

The datasets presented in this study can be found in online repositories. The names of the repository/repositories and accession number(s) can be found at: https://www.ncbi.nlm.nih.gov/, PRJNA1224288.

## Supplementary material

SUPPLEMENTARY FIGURE S1 Description of the localities. **(A–D)** Trends of pollutant individual recordings over the course of 5 years recorded by AQM stations: **(A)** Overall, **(B)** Hos, **(C)** Bai, **(D)** Mri.

SUPPLEMENTARY FIGURE S2 Compositional differences in the gut microbiome. **(A,B)** Relative abundances of top 20 taxa sorted based on abundance sum sample level granularity: **(A)** phylum level, **(B)** genus level.
