# Supplementary material for: Ecological associations between ambient air pollution, gut microbiome composition, and metabolic markers in urban populations of central India
Source: Front Public Health. 2026 Sep 14;14:1834285. doi: 10.3389/fpubh.2026.1834285 (PMC13617079; doi:10.3389/fpubh.2026.1834285)
Supplement: Supplementary file 1 [file Data_Sheet_1.pdf]

**Table S1:** Descriptive statistics of ambient air pollutant concentrations (in µg/m³) across the study period.

| **Index** | **PM10** | **PM2.5** | **NOx** | **SO2** |
|---|---|---|---|---|
| Count | 1303.000 | 996.000 | 1295.000 | 1295.000 |
| Mean | 129.380 | 55.329 | 15.462 | 6.366 |
| Median | 126.1 | 51.85 | 14.3 | 5.6 |
| Std | 54.351 | 23.961 | 8.171 | 4.034 |
| Min | 47.300 | 12.000 | 2.000 | 2.000 |
| 25% | 83.900 | 40.100 | 9.900 | 3.100 |
| 50% | 126.100 | 51.850 | 14.300 | 5.600 |
| 75% | 164.200 | 67.700 | 20.350 | 8.800 |
| Max | 543.400 | 233.300 | 55.700 | 27.500 |
| CV | 42.009 | 43.307 | 52.842 | 63.376 |
| Skewness | 1.281 | 1.401 | 0.763 | 1.188 |
| Kurtosis | 4.735 | 5.683 | 0.961 | 1.980 |
| K-S test P | 0.000 | 0.000 | 0.000 | 0.000 |

CV (coefficients of variation in %), K-S test P (P values from Kolmogorov–Smirnov test), std (standard deviation)

**Table S2:** Results of descriptive PCA considering the pollutant, prevalence and microbiome data. Explained variance ratio: [0.65626713, 0.34373287].

| **Loadings:** | **PC1** | **PC2** |
|---|---|---|
| Prevalence_T2D (%) | -0.20236 | 0.070287 |
| Prevalence_PD (%) | 0.192444 | -0.11142 |
| Avg PM10 | 0.199084 | 0.086312 |
| Avg PM2.5 | 0.206129 | -0.04471 |
| Avg Nox | 0.08263 | -0.26474 |
| Avg SO2 | 0.206377 | 0.042476 |
| HbA1c | 0.119193 | -0.23664 |
| FBS | -0.05482 | -0.27818 |
| Insulin | -0.17845 | -0.14942 |
| Body Mass Index | -0.16865 | -0.16975 |
| Waist to Hip Ratio | -0.12674 | -0.22903 |
| Waist to Height Ratio | -0.17717 | -0.15229 |
| QUICKI | 0.162273 | 0.181234 |
| HOMA-IR | -0.18626 | -0.12994 |
| HOMA-B | -0.19675 | 0.09599 |
| Bifidobacterium | 0.183792 | 0.136485 |
| Lactobacillus | -0.11843 | 0.237365 |
| Prevotella_9 | -0.20842 | 0.013677 |
| Streptococcus | 0.107424 | -0.24716 |
| Dialister | 0.029256 | 0.285459 |
| Faecalibacterium | 0.206235 | 0.04377 |
| Catenibacterium | -0.18368 | 0.136786 |
| Agathobacter | 0.206174 | -0.04431 |
| Blautia | -0.06774 | 0.272689 |
| Escherichia/Shigella | 0.207632 | 0.028496 |
| Collinsella | -0.154 | 0.194523 |
| Holdemanella | 0.208199 | -0.01902 |
| Dorea | -0.20099 | -0.07742 |
| Clostridium_sensu_stricto_1 | 0.201606 | 0.074297 |
| Romboutsia | 0.208442 | 0.012985 |
| Bacteroides | -0.20706 | 0.035595 |
| Subdoligranulum | -0.12831 | 0.227348 |
| Alloprevotella | 0.001152 | 0.288303 |
| Megasphaera | -0.20809 | 0.021259 |
| Others | -0.03849 | -0.28336 |

**Table S3:** Baseline demographic, lifestyle, and metabolic status characteristics of study participants across the three study locations (Bai, Hos, and Mri).

| Variable | Bai | Hos | Mri |
|---|---|---|---|
| Age | 49.53 ± 9.73 | 50.92 ± 7.33 | 48.78 ± 11.02 |
| SBP | 139.47 ± 17.24 | 131.17 ± 17.41 | 123.33 ± 10.78 |
| DBP | 85.61 ± 10.90 | 82.33 ± 12.52 | 80.00 ± 8.70 |
| PP | 53.85 ± 15.98 | 48.83 ± 9.14 | 43.33 ± 7.58 |
| FBS | 133.97 ± 64.52 | 120.46 ± 33.52 | 112.66 ± 35.41 |
| HbA1c (DCCT) | 7.44 ± 2.32 | 6.77 ± 1.23 | 6.66 ± 1.37 |
| Height (cm) | 160.17 ± 10.39 | 159.33 ± 9.33 | 168.22 ± 10.17 |
| Weight (kg) | 67.44 ± 12.41 | 69.30 ± 13.24 | 68.27 ± 13.53 |
| Waist circumference (cm) | 94.96 ± 9.96 | 96.36 ± 10.01 | 92.05 ± 9.48 |
| Hip circumference (cm) | 99.60 ± 10.08 | 100.14 ± 9.18 | 97.05 ± 5.13 |
| BMI | 26.44 ± 5.38 | 27.17 ± 3.99 | 24.12 ± 4.12 |
| WHR | 0.95 ± 0.05 | 0.96 ± 0.05 | 0.94 ± 0.06 |
| WHtR | 0.59 ± 0.07 | 0.60 ± 0.05 | 0.54 ± 0.05 |
| Sex: M | 27 (43.5%) | 12 (50.0%) | 7 (77.8%) |
| Sex: F | 35 (56.5%) | 12 (50.0%) | 2 (22.2%) |
| Condition: T2D | 33 (53.2%) | 17 (70.8%) | 4 (44.4%) |
| Condition: Ctrl | 11 (17.7%) | 3 (12.5%) | 2 (22.2%) |
| Condition: PD | 18 (29.0%) | 4 (16.7%) | 3 (33.3%) |
| Diet Choice: V | 19 (51.4%) | 10 (55.6%) | 3 (50.0%) |
| Diet Choice: N | 18 (48.6%) | 8 (44.4%) | 3 (50.0%) |
| Smoking: No | 44 (75.9%) | 19 (82.6%) | 5 (55.6%) |
| Smoking: Yes | 14 (24.1%) | 4 (17.4%) | 4 (44.4%) |
| Alcohol: Yes | 21 (36.8%) | 4 (17.4%) | 1 (11.1%) |
| Alcohol: No | 36 (63.2%) | 19 (82.6%) | 8 (88.9%) |
| SES: High | 9 (16.4%) | 11 (52.4%) | 3 (33.3%) |
| SES: Middle | 19 (34.5%) | 6 (28.6%) | 1 (11.1%) |
| SES: Low | 27 (49.1%) | 4 (19.0%) | 5 (55.6%) |
| Physical Activity: No | 26 (48.1%) | 7 (33.3%) | 6 (66.7%) |
| Physical Activity: Yes | 28 (51.9%) | 14 (66.7%) | 3 (33.3%) |

Continuous variables are presented as mean ± standard deviation (SD). Categorical variables are presented as number of participants and percentage [n (%)]. Percentages were calculated after excluding missing values for each variable. Diet categories include vegetarian (V) and non-vegetarian (N). SES indicates socioeconomic status.

## Exploratory PCA of Microbiome, Clinical, and Pollution Data

PCA was performed on city-level averages of microbial genera, metabolic parameters, and air pollution variables. The first two components explained 100% of the variance (PC1 = 65.6%, PC2 = 34.4%). Loadings indicated that PC1 was primarily associated by air pollution indicators ($PM_{2.5}$, $PM_{10}$, $SO_2$) and certain microbial genera (e.g., *Faecalibacterium*, *Agathobacter*, *Bacteroides*), whereas PC2 was associated with metabolic measures and other microbial taxa. Because only three city-level observations were included, these results are mathematically constrained; and should be interpreted as exploratory and descriptive rather than inferential, highlighting potential trends rather than robust biological patterns.
